# Anti‐saccadic task can distinguish between patients at risk of developing Alzheimer’s disease and cognitively unimpaired patients in a memory clinic

**DOI:** 10.1002/alz.090297

**Published:** 2025-01-03

**Authors:** Zuzana Wallin Ištvánfyová, Mattias Nilsson, Göran Hagman, Miia Kivipelto

**Affiliations:** ^1^ Karolinska Institutet, Stockholm Sweden; ^2^ Karolinska University Hospital, Stockholm Sweden

## Abstract

**Background:**

Early detection is crucial for alleviating Alzheimer’s disease (AD) burden. At present, assessment for early detection of AD is time‐consuming, costly, and often invasive. In recent years, various eye‐tracking methodologies have emerged, and they show promising results in detecting persons at risk of developing AD dementia (ADD). This study aims at identifying suitable anti‐saccadic measures to detect people with early AD.

**Method:**

Preliminary data from 21 cognitively impaired and 45 cognitively unimpaired individuals was collected from a memory clinic (Karolinska University Hospital, Sweden). The data included results from an anti‐saccadic task, neuropsychological tests battery, demographic information, cerebrospinal fluid markers (CSF), and AI‐computed volumetry from magnetic resonance imaging. Median values were calculated on anti‐saccadic measures with the strongest associations to cognition, CSF, and volumetry. The identified measures were proportion of errors (PoE) and latency of a correction saccade (LoCS). Subsequently, the clusters of high and low performers were compared using the Mann‐Whitney U test.

**Result:**

Both PoE and LoCS were able to distinguish between high and low atrophy grade in all cortical brain regions with the highest effect size found in the right cerebral lobe for PoE (U = 266.00, d = 1.36, p < .001) and the right parietal lobe for LoCS (U = 227.50, d = 1.234, p < .002). PoE and LoCS could also distinguish between high and low cognitive function, with the highest effect size for LoCS in verbal delayed recall (U = 210.50, d = 1.061, p < .001) and visual immediate recall (U = 217.00, d = 1.027, p < .001). We did not find any significant differences in the CSF between the clusters.

**Conclusion:**

A brief anti‐saccadic task was able to distinguish between individuals with and without brain atrophies, with PoE capturing atrophy grade with largest effect size. Similarly, the task distinguished individuals with better and worse cognition, with LoCS resulting in largest effect sizes. Our results indicate that both PoE and LoCS have a potential to distinguish individuals at risk of ADD from cognitively unimpaired individuals. Furthermore, the lack of CSF association raises questions about chronology of pathological processes involved in AD.

